# Nurses’ attitudes toward patient death and coping strategies: a cross-sectional study in Poland

**DOI:** 10.3389/fpubh.2025.1670848

**Published:** 2025-11-04

**Authors:** Anna Maria Cybulska, Izabela Czeryna, Aleksandra Derezińska, Marta Nowak, Lilianna Majkowska, Daria Schneider-Matyka, Elżbieta Grochans, Kamila Rachubińska

**Affiliations:** ^1^Department of Nursing, Faculty of Health Sciences, Pomeranian Medical University in Szczecin, Szczecin, Poland; ^2^Independent Public Provincial Hospital in Szczecin, Szczecin, Poland; ^3^Department of Diabetology and Internal Diseases, Pomeranian Medical University in Szczecin, Szczecin, Poland

**Keywords:** attitudes toward death, death anxiety, death and dying, end of life care, nurse, nursing care

## Abstract

**Introduction:**

Nurses working in end-of-life care are frequently exposed to patient death, which shapes both their attitudes toward death and the coping strategies they adopt. This study aimed to explore nurses’ attitudes toward death and the coping mechanisms they employ in the hospital setting.

**Methods:**

Data were collected through a diagnostic survey incorporating a self-designed questionnaire and standardized instruments: the Death Attitudes Profile Questionnaire and the MINI-COPE Scale.

**Results:**

The study included 315 nurses (85.7% women) with a mean age of 40.5 years. Most participants lived in towns of up to 100,000 residents (70.1%) and held a master’s degree (53.3%). The predominant attitude toward death was Natural Acceptance (5.40 ± 0.97 point). The most commonly used coping strategies were Preoccupation with Other Activities (1.98 ± 0.75 point), Active Coping (1.88 ± 0.69 point), and Planning (1.87 ± 0.77 point). Greater exposure to patient death was associated with higher levels of Natural Acceptance and Escape Acceptance, alongside lower levels of Death Avoidance. Negative emotions most frequently reported were sadness (62.9%), compassion (57.5%), and helplessness (47.0%), underscoring the emotional burden of end-of-life care.

**Conclusion:**

Nurses frequently experience negative emotional responses to patient death, emphasizing the need for accessible psychological support. Sociodemographic and professional factors significantly influence both attitudes toward death and stress-coping strategies, highlighting the importance of targeted interventions to strengthen resilience among nursing staff in end-of-life care.

## 1 Introduction

Death represents one of humanity’s most profound existential concerns. With advancements in medicine and the development of modern intensive care, which have significantly increased survival rates, the distinction between life and death has become less defined, and the dying process has been prolonged. Death is now viewed as a transitional phase, marking the cessation of life (1). Despite these technological innovations, predicting the exact moment of death remains elusive, underscoring the critical importance of ensuring the peace and dignity of patients as they approach the end of life. This includes addressing their physical, mental, social, emotional, spiritual, and existential needs to provide comprehensive comfort during this final stage (2).

Attitudes toward this phenomenon can be either negative or positive, shaped by emotional and psychological responses to death (3). Attitudes toward death vary and depend on many factors such as experience, knowledge and individual beliefs. Each person approaches the subject in his or her own way, and attitudes may change as time passes and new experiences are gained. Contemporary approaches to death vary depending on context and individual beliefs. Some people tame the process of dying and prepare for it, accepting death as a natural phenomenon, while others feel fear or choose an escape attitude toward death (4).

The Death Attitude Profile-Revised (DAP-R) conceptualizes five dimensions of attitudes toward death: fear of death, death avoidance, natural acceptance, approach acceptance, and escape acceptance (5, 6). Natural acceptance reflects viewing death as a normal, inevitable part of life, often linked to cultural or religious beliefs and associated with adaptive coping strategies. In contrast, escape acceptance frames death as a release from suffering, which may be understandable in palliative care contexts but can also indicate emotional burden or professional burnout.

The Terror Management Theory (TMT) (7) posits that awareness of one’s mortality triggers existential anxiety, which individuals manage through adherence to cultural worldviews and self-esteem maintenance. The Meaning Management Theory (MMT) (8) emphasizes that acceptance-based attitudes toward death serve as a way to preserve life meaning and purpose in the face of mortality. Integrating these theoretical frameworks allows a deeper understanding of why certain acceptance types prevail among nurses and what psychological mechanisms may underpin them.

In Poland, the nursing profession operates within a healthcare system characterized by significant workforce shortages and one of the lowest nurse-to-population ratios in the European Union—approximately 5.1 nurses per 1,000 inhabitants compared to the OECD average of about 9 per 1,000 (9). The profession is also highly feminized, with women comprising over 90% of the nursing workforce (10). These demographic characteristics, combined with systemic challenges such as high workloads, multiple job holding, and limited access to structured postgraduate training in palliative and end-of-life care (11), shape nurses’ experiences and their preparedness to care for dying patients. In addition, the cultural and religious context—Poland being predominantly Roman Catholic—may reinforce attitudes such as natural acceptance of death, while high exposure to death in clinical practice may contribute to the development of coping strategies that prioritize emotional detachment or acceptance. Studies conducted in various cultural contexts demonstrate that nurses’ attitudes toward death are shaped not only by professional factors but also by cultural norms and religious beliefs. For example, Cardoso et al. (12) found that Portuguese nurses often exhibited fear and avoidance in response to death, whereas in Brazil, Kovacevic et al. (13) reported higher acceptance linked to Catholic traditions. In South Korea, Kim and Kim (14) observed that Confucian and Buddhist influences promoted a more fatalistic acceptance of death, while in Iran, Banner et al. (15) noted that Islamic beliefs fostered approach acceptance. These differences highlight the importance of cultural and religious frameworks in shaping death-related attitudes, making cross-cultural comparisons essential.

Due to the nature of their work, nurses frequently care for patients in critical condition or nearing the end of life. The death of even an unfamiliar patient can be a stressful experience, impacting their emotional well-being (16). Therefore, the nursing profession is considered one of the most stressful and demanding, as well as taxing professions. The type of work performed forces nurses to have the ability to cope with stress, with difficult situations, as well as to make quick decisions (17). Working under pressure, nurses’ personalities and individual experiences affect their attitudes toward death, and thus can affect the quality of medical care and patient safety (18, 19). Work-related stress can negatively affect many aspects of nurses’ lives and contribute to many health complications, as well as burnout syndrome (20). Nurses, in order to protect themselves from the effects of negative attitudes toward patient death (e.g., fear of death), may choose strategies to protect themselves from these emotions (e.g., denial of feelings), which can be a defense mechanism against the effects associated with accompanying a patient through dying and death (21, 22).

Nurses’ attitudes toward caring for a dying patient have a significant impact on the quality of care provided. Caring for a critically ill patient can cause anxiety and emotional exhaustion, which in turn can lead to professional burnout (23). Accompanying a dying patient affects nurses’ lives through changes in thinking, values, roles, beliefs, codes of conduct or perceptions of their professional identity (24). Most nurses remember the first death of a patient because it is a difficult experience laden with many emotions such as grief, sadness, fear or helplessness. The attitudes nurses adopt toward a patient’s death depend on the age of the dying person, the manner of death and the relationship the nurse established while the deceased was alive (21). Through the lack of these skills, the nurse feels a lack of job satisfaction, higher levels of stress, which compromises the quality of care (25). While nurses’ attitudes toward death and coping strategies have been studied internationally, there is a scarcity of recent data from Poland, particularly in the post-COVID-19 healthcare context. This study is unique in its concurrent examination of death attitudes and stress coping strategies in a representative group of nurses in the West Pomeranian Voivodeship, integrating these psychological constructs with sociodemographic and professional variables. Additionally, unlike many previous studies focused on a single clinical setting, our sample included nurses from diverse hospital departments, enabling a broader perspective on factors influencing end-of-life care. The findings contribute new insights into how cultural, religious, and systemic factors shape nurses’ experiences, thus filling an important gap in the nursing literature. Therefore, the aim of this study is to analyze attitudes toward death among the nursing staff in hospital. We formulated the following research questions:

- What are the predominant attitudes of hospital nurses toward patient death?
- How do selected sociodemographic variables (e.g., age, gender, educational attainment) and professional characteristics (e.g., years of clinical experience, hospital ward type) affect nurses’ attitudes toward patient death?
- What is the relationship between nurses’ stress-coping strategies and their attitudes toward patient death?

## 2 Materials and methods

### 2.1 Course and organization of the study

The research was conducted using a diagnostic survey method, employing a questionnaire as the primary data collection tool.

Inclusion criteria were: (1) current employment as a registered nurse in a hospital setting in the West Pomeranian Voivodeship, (2) minimum of one year of professional experience, and (3) direct patient care responsibilities. Nurses from various units, including surgical, conservative care, intensive care, oncology, palliative care, and emergency departments, were included. Data were collected from X hospitals of varying sizes (district, provincial, and university hospitals), ensuring diversity of clinical settings.

Exclusion criteria were: (1) nurses employed outside the West Pomeranian Voivodeship, (2) nurses with less than one year of professional experience, (3) nurses not engaged in direct patient care (e.g., administrative, managerial), (4) nursing students (5) refusal to provide informed consent.

Respondents were acquainted with the subject matter and purpose of the study, and informed that the data obtained would be used only for the purposes of the scientific study, and that participation in the study was voluntary and anonymous. Participants were informed that they could withdraw their consent to participate in the study at any stage, without giving a reason. The return of properly completed questionnaires was tantamount to consent to the study.

The sample size was calculated based on the total population of approximately 14,000 registered nurses in the West Pomeranian Voivodeship, according to the 2023 report of the Supreme Chamber of Nurses and Midwives (10). We applied Cochran’s formula for finite populations (26).


N×Z^2×p×(1−p)e^2×(N−1)+Z^2×p×(1−p)

Assuming a 95% confidence level (*Z* = 1.96), a maximum margin of error (e) of 0.06, and an estimated proportion (*p*) of 0.5. This yielded a minimum required sample size of 260 nurses. To account for potential non-response, 340 questionnaires were distributed.

### 2.2 Research methods and tools used

The study employed the diagnostic survey method. The diagnostic survey method refers to a structured approach for collecting standardized data from a defined population using validated instruments, enabling the description and analysis of specific phenomena—in this case, nurses’ attitudes toward death and coping strategies.

In the course of the study, the author’s survey questionnaire and standardized research tools were used:

- **The Death Attitudes Profile Revisited (DAP-R-PL)** in the revised version by PTP Wong, GT Reker, G. Gesser. The questionnaire consists of 32 statements relating to various attitudes toward death. Comparison of the average scores obtained on the individual scales of the DAP-R-PL questionnaire for the entire study group of respondents makes it possible to determine the dominant attitude toward death in the study group, such as: fear of death; death avoidance; natural acceptance of death; theological acceptance of death; escape avoidance. Each statement is scored from 1 to 7, depending on the answer chosen. The arithmetic mean is calculated from the sum of the scores obtained for all questions in a given scale from all respondents, and the value obtained is a measure of the prevalence of a given attitude in the study group. Comparison of the obtained indices for a given scale indicates which attitude among the five included in the questionnaire dominates among respondents. Cronbach’s alpha coefficient for the five DAP-R-PL factors ranged between *α* = 0.76 and α = 0.86 (15).
- **MINI-COPE** – The Mini-COPE Stress Coping Inventory by C. S. Carver, in a Polish adaptation by Zygfryd Juczynski and Nina Oginska-Bulik, is a shortened version of the Multidimensional Coping Inventory. It is a questionnaire for assessing strategies for coping with stress, which consists of 28 statements, making a total of 14 strategies such as Acceptance, Affective coping, Blame, Seeking emotional support, Seeking instrumental support, Planning, Positive reevaluation, Sense of humor, Howling, Denial, Preoccupation with something else, Cessation of activities, Use of psychoactive substances, Turning to religion. The aforementioned strategies can be grouped into three styles of coping with stress: evasive, emotion-focused and problem-focused. The MINI-COPE inventory consists of 28 items rated on a 4-point Likert scale (0 – “I have not been doing this at all” to 3 – “I’ve been doing this a lot”), grouped into 14 coping strategies. For each strategy, the score is calculated by summing the responses to the two relevant items; higher scores indicate more frequent use of the coping strategy in stressful situations (27). The Polish adaptation of the Mini-COPE inventory (5) has demonstrated good psychometric properties in previous validation studies, with Cronbach’s *α* values for the subscales ranging from 0.62 to 0.89. In the present study, the internal consistency of the Mini-COPE was also satisfactory, with Cronbach’s α coefficients ranging from 0.65 to 0.88 across the subscales, indicating acceptable to high reliability (27).
- In addition to the two standardized instruments, participants completed a self-designed sociodemographic questionnaire developed by the authors for the purposes of this study. This form included questions on age, gender, place of residence, marital status, education, professional specialization, years of professional experience, and the hospital unit in which the nurse was employed.

### 2.3 Ethical aspect

All procedures performed involving human participants were in accordance with the ethical standards of the institutional research committee and with the Helsinki Declaration. The study was approved by the [Anonymous] Bioethics Committee (KB-0012/219/06/16). Our study was conducted taking into account ethical considerations. Informed consent was required, and participation in the study was voluntary. Moreover, the participants were assured of anonymity and confidentiality and were free to withdraw from the study at any moment.

### 2.4 Statistical analysis

Data were collected using Microsoft Excel and analyzed in STATISTICA 10 PL. Descriptive statistics (mean, standard deviation, median, quartiles, minimum, and maximum) were calculated for quantitative variables, while frequencies and percentages were used for qualitative variables. Due to the non-normal distribution of measurable variables, as determined by the Shapiro–Wilk test, non-parametric tests were applied: the Mann–Whitney U test for comparisons between two groups and the Kruskal–Wallis test for three or more groups, including post-hoc multiple comparisons. Spearman’s rank correlation coefficient was used for variables not meeting the assumptions of normality, whereas Pearson’s correlation coefficient was applied for normally distributed variables. A significance level of 0.05 was used; *p*-values below this threshold were considered statistically significant.

## 3 Results

### 3.1 Influence of sociodemographic and work-related variables on nurses’ attitudes toward patient death according to DAP-R-PL

A total of 315 nurses participated in the survey, with the vast majority of respondents (85.7%) being women. The average age was 40.5 years (SD = 10.7). The dominant group was: living in a city of up to 100,000 residents (70.1%), in a formal relationship (39%), with a master’s degree (53.3%) and specialization (58.1%). The average length of service was 17.9 years (SD = 11.4). Most respondents worked: in a single workplace (51.1%) and in a surgical department (56.5%). Respondents were asked to indicate the unit where they spend the majority of their working hours; some overlap in patient profiles between units is possible. (Table 1).

**Table 1:** Nurse characteristics.

Variable		Female (*n* = 270)		Male (*n* = 45)		Total (*n* = 315)	
		*n*	%	*n*	%	*n*	%
Place of residence	Rural	17	6.3	10	22.2	27	8.6
	City up to 100 thousand inhabitants.	201	74.4	20	44.5	221	70.1
	City above 100 thousand residents	52	19.3	15	33.3	67	21.3
Marital status	In a formal relationship	114	42.2	9	20.0	123	39.1
	In an informal relationship	94	34.8	23	51.1	117	37.1
	Single/unmarried	62	23.0	13	28.9	75	23.8
Education	Medical school	39	14.4	13	28.9	52	16.6
	Higher education - bachelor’s degree	84	31.1	10	22.2	94	29.8
	Higher education - master’s degree	147	54.5	22	48.9	169	53.6
Specialization	Yes	155	57.4	28	62.2	183	58.1
	No	115	42.6	17	37.8	132	41.9
Working in one place	Yes	152	56.3	9	20.0	161	51.1
	No	118	43.7	36	80.0	154	48.9
Place of work*	Surgical Unit (e.g., general surgery, orthopedic surgery)	148	54.8	30	66.7	178	56.5
	Primary Medical Care	64	23.7	22	48.9	86	27.3
	Conservative Care Unit (e.g., internal medicine, cardiology)	45	16.7	11	24.4	56	17.8
	Hospice	21	7.8	6	13.3	27	8.6
	Pediatrics	20	7.4	2	4.4	22	7.0
	Intensive Care Unit (ICU)	20	7.4	4	8.9	24	7.6
	Other	62	22.9	7	15.5	69	21.9

n, number of respondents; ^*^Respondents were asked to indicate the unit where they spend the majority of their working hours; some overlap in patient profiles between units is possible.

More than half of the respondents remembered the first death of a patient (57.5%). The emotions most frequently experienced by nurses in connection with a patient’s death were sadness (62.9%), compassion (57.5%), helplessness (47.0%), grief (45.7%), and a sense of injustice (43.5%).

Interpreting the data on the frequency of experiencing a patient’s death, it was found that nurses most often experienced a patient’s death several times a month (34.3%). Other respondents experienced patient death: once a month (18.4%), several times a year (26.3%), and annually (5.7%). In contrast, death was experienced once a week by 15 respondents (4.8%), and several times a week by 19 respondents (6.0%). Only 14 respondents (4.4%) reported never having experienced a patient’s death.

The majority of respondents (63.8%) reported that the death of a patient did not impact the quality of their work. However, 24.8% felt that a patient’s death did affect their work, while 11.4% of respondents had no opinion on the matter. Nurses identified several factors influencing their attitudes toward patient death, with the patient’s age being the most significant factor (77.5%), followed by the type of death (62.2%) and the relationship with the patient (59.7%). In contrast, 31.1% of respondents believed that the relationship with the patient’s family played a role in shaping their attitudes toward the death. When it came to coping mechanisms, respondents indicated that the most effective methods for managing the emotional impact of patient death were support from a psychologist (62.2%), longer work experience (61.3%), and frequent exposure to patient death (54.9%). On the other hand, only 15.2% of respondents believed that relaxation techniques were a useful approach for managing stress. Nearly half of the respondents (49.5%) were unaware of whether their employer provided psychological support for staff struggling to cope with the death of a patient. Only 13.7% of women reported that their workplace offered such support, while 38.7% of respondents did not have access to employer-provided psychological assistance.

### 3.2 Analysis of nurses’ attitudes toward patient death according to DAP-R-PL and stress coping strategies according to MINI-COPE

Based on the interpretation of the DAP-R-PL questionnaire, it was shown that the attitudes Natural Acceptance (5.40 ± 0.97pts) and Escape Acceptance (5.09 ± 1.01pts) predominated among respondents. The attitudes Approach Acceptance (4.08 ± 1.39pts) and Death Avoidance (4.17 ± 1.25pts) were the least common among respondents (Table 2).

**Table 2:** Descriptive statistics of attitudes toward death on the DAP-R-PL and MINI-COPE stress coping strategies scales.

Variable		M ± SD	Me	Min.- Max
DAP-R-PL	Fear of death	4.72 **±** 0.97	4.86	1.43–6.86
	Death avoidance	4.17 **± 1**.25	4.20	1.00–7.00
	Natural acceptance	5.40 **±** 0.97	5.60	1.40–7.00
	Approach acceptance	4.08 **±** 1.39	4.00	1.00–7.00
	Escape acceptance	5.09 **±** 1.01	5.20	1.00–7.00
MINI-COPE	Active coping	1.88 **±** 0.69	2.00	0.00–3.00
	Planning	1.87 **±** 0.77	2.00	0.00–3.00
	Positive reframing	1.68 **±** 0.64	1.50	0.00–3.00
	Acceptance	1.84 **±** 0.62	2.00	0.00–3.00
	Humor	0.97 **±** 0.84	1.00	0.00–3.00
	Religion	1.16 **±** 1.18	1.00	0.00–3.00
	Use of emotional support	1.79 **±** 0.53	2.00	0.00–3.00
	Use of instrumental support	1.73 **±** 0.55	2.00	0.00–3.00
	Self-distraction	1.98 **±** 0.75	2.00	0.00–3.00
	Denial	1.31 **±** 0.74	1.50	0.00–3.00
	Venting	1.71 **±** 0.70	2.00	0.00–3.00
	Substance use	0.96 **±** 1.05	1.00	0.00–3.00
	Behavioral disengagement	1.35 **±** 0.83	1.00	0.00–3.00
	Self-blame	1.11 **±** 0.67	1.00	0.00–3.00

M ± SD, Mean ± Standard deviation; Me, median; Min.–Max., minimum – maximum.

An analysis of the MINI-COPE questionnaire responses indicated that the most prevalent stress coping strategies among the participants were: Preoccupation with Other Activities (1.98 ± 0.75), Active Coping (1.88 ± 0.69), Planning (1.87 ± 0.77), and Acceptance (1.84 ± 0.62). Conversely, the least frequently employed strategies included: Use of Psychoactive Substances (0.96 ± 1.05), Sense of Humor (0.97 ± 0.84), Self-Blame (1.11 ± 0.67), and Turning to Religion (1.16 ± 1.18) (Table 2).

The study analyzed the relationship between nurses’ choice of attitudes toward patient death according to the DAP-R-PL and selected sociodemographic variables (age, gender, place of residence, marital status). The data analysis showed a statistically significant relationship between age and nurses’ attitudes toward patient death according to the DAP-R-PL. The study noted a weak negative correlation between the subjects’ age and fear of death (*p* = 0.0018) once a weak positive correlation between age and Approach Acceptance (*p* = 0.0069). There were no statistically significant correlations between age and other attitudes toward death according to the DAP-R-PL (Table 3).

**Table 3:** Relationship of age and attitudes of respondents toward death according to DAP-R-PL.

DAP-R-PL	Age (years)	
	*r*	*p*
Fear of death	−0.175	0.0018
Death avoidance	−0.051	0.3658
Natural acceptance	−0.076	0.1777
Approach acceptance	0.152	0.0069
Escape acceptance	0.050	0.3785

*r*, Pearson correlation coefficient; *p*, significance level.

The study showed statistically significant differences regarding attitudes toward patient death according to the DAP-R-PL according to the gender of the subjects. Significant differences were observed regarding attitudes toward death: Fear of death (*p* = 0.0020), Natural Acceptance (*p* = 0.0059) and Approach Acceptance (*p* = 0.0364). This means that the above attitudes toward death were used more often by women than men. For the remaining attitudes toward death according to the DAP-R-PL, there were no statistically significant differences between men and women (Table 4).

**Table 4:** Attitudes toward death according to DAP-R-PL according to sociodemographic variables.

Variable		Fear of death	Death avoidance	Natural acceptance	Approach acceptance	Escape acceptance
		M ± SD	M ± SD	M ± SD	M ± SD	M ± SD
Gender	Female	4.79 ± 0.96	4.22 ± 1.23	5.47 ± 0.92	4.15 ± 1.40	5.11 ± 1.00
	Male	4.34 ± 0.90	3.88 ± 1.33	5.00 ± 1.14	3.65 ± 1.26	5.01 ± 1.09
	*Z*	3.09	1.66	2.75	2.09	1.18
	*p*	0.002	0.0976	0.0059	0.0364	0.2370
Place of residence	Rural	4.26 ± 1.23	3.99 ± 1.50	5.04 ± 1.13	4.31 ± 1.30	5.07 ± 1.20
	City ≤ 10,000 residents	4.63 ± 1.09	4.18 ± 1.26	5.36 ± 1.06	4.38 ± 1.44	5.28 ± 1.08
	City 10–100,000 residents	4.89 ± 0.82	4.07 ± 1.16	5.68 ± 0.86	3.84 ± 1.47	5.26 ± 0.87
	City > 100,000 residents	4.59 ± 1.00	4.48 ± 1.34	4.90 ± 0.81	4.31 ± 1.08	4.53 ± 1.00
	*H*	10.70	6.26	45.20	12.40	32.50
	*p*	0.0132	0.0995	<0.0001	0.0062	<0.0001
Marital status	In a formal relationship	4.72 ± 1.01	4.33 ± 1.18	5.27 ± 0.94	4.57 ± 1.36	4.98 ± 1.04
	In an informal relationship	4.71 ± 0.86	4.03 ± 1.21	5.53 ± 0.93	3.72 ± 1.36	5.21 ± 1.00
	Single/Unmarried	4.75 ± 1.06	4.13 ± 1.40	5.40 ± 1.05	3.85 ± 1.29	5.10 ± 0.98
	*H*	0.08	3.53	6.00	25.60	5.60
	*p*	0.9593	0.1713	0.0498	<0.0001	0.0607
Education	Medical school*	4.32 ± 1.13	4.07 ± 1.32	5.19 ± 1.17	4.33 ± 1.48	5.38 ± 1.22
	Bachelor’s degree	4.59 ± 1.00	4.30 ± 1.30	5.10 ± 0.95	4.31 ± 1.21	4.64 ± 0.97
	Master’s degree	4.92 ± 0.84	4.13 ± 1.20	5.63 ± 0.85	3.88 ± 1.44	5.26 ± 0.88
	*H*	17.04	1.64	25.85	9.22	32.33
	*p*	0.0002	0.4408	<0.0001	0.0099	<0.0001

*H*, value of Kruskal-Wallis test; *z*, value of Mann–Whitney U test; *p*, significance level; *Medical school, refers to post-secondary vocational nursing education, typically preparing graduates for licensure as registered nurses.

Analysis of the data showed statistically significant differences regarding nurses’ attitudes toward patient death according to DAP-R-PL depending on the respondents’ place of residence. The differences were related to attitudes toward death: Anxiety of death (*p* = 0.0132), Natural acceptance of death (*p* < 0.0001), Approach Acceptance (*p* = 0.0062), Escape Acceptance (*p* < 0.0001). It was shown that Fear of death (*p* = 0.0348) and Natural Acceptance (*p* = 0.0116) were used more often by people living in a city of 10–100,000 residents than by rural residents. In the case of Approach Acceptance (*p* = 0.0062), it was those living in a city of more than 100,000 residents who were more likely to use this attitude than respondents from cities of 10–100,000 residents. It was also shown that Escape Acceptance was more often chosen by those from rural areas than those from cities with more than 100,000 residents (*p* = 0.0468) (Table 4).

Analysis of the data showed statistically significant relationships between marital status and nurses’ attitudes toward patient death according to the DAP-R-PL. The study noted significant differences between respondents with different marital status for attitudes toward death: Natural Acceptance (*p* = 0.0498), Approach Acceptance (*p* < 0.0001). Analysis of the data showed statistically significant differences regarding nurses’ attitudes toward patient death according to DAP-R-PL according to respondents’ marital status. It was shown that Natural Acceptance was more often used by those living in an informal relationship than those in a formal relationship (*p* = 0.0477) In the case of Approach Acceptance, it was those living in a formal relationship who chose this attitude toward death more often than those living in an informal relationship (*p* < 0.0001) and then those who were single (*p* = 0.0009) (Table 4). Analysis of the data showed statistically significant differences in attitudes toward patient death according to the DAP-R-PL depending on the respondents’ education. The study showed statistically significant differences between education and Fear of death (*p* = 0.0002), Natural Acceptance (<0.0001), Approach Acceptance (*p* = 0.0099) and Escape Acceptance (*p* < 0.0001) (Table 4).

The study analyzed the relationship between nurses’ choice of attitudes toward patient death according to the DAP-R-PL and selected work-related variables (having a specialty, length of service and frequency of experiencing patient death).

Analysis of the data showed statistically significant differences in nurses’ attitudes toward patient death according to the DAP-R-PL depending on specialty possession. These were differences in attitudes toward death: Death Avoidance (*p* < 0.0001), Natural Acceptance (*p* < 0.0001), Escape Acceptance (*p* = 0.009). Those with specialization were more likely to use Naturl Acceptance and Escape Acceptance than those without specialization. In contrast, those without specialization were more likely to use the Death Avoidance attitude than those with specialization. No statistically significant differences were shown for the other attitudes (Table 5).

**Table 5:** Attitudes toward death according to DAP-R-PL according to work-related variables.

Variable			DAP-R-PL				
			Fear of death	Death avoidance	Natural acceptance	Approach acceptance	Escape acceptance
Specialization	Yes	M ± SD	4.64 ± 1.01	3.94 ± 1.23	5.55 ± 0.95	4.02 ± 1.49	5.2 ± 1.03
	No	M ± SD	4.83 ± 0.9	4.49 ± 1.22	5.18 ± 0.95	4.17 ± 1.25	4.95 ± 0.97
	*Z*		−1.37	−3.99	3.96	−1.23	2.62
	*p*		0.17	<0.0001	<0.0001	0.22	0.009
Seniority in the profession (years) (*n* = 300)	*r*		−0.14	−0.09	0.01	0.13	0.09
	*p*		0.02	0.14	0.85	0.02	0.13
Frequency of experiencing patient death (*n* = 315)	*r*		0.03	−0.223	0.231	−0.137	0.233
	*p*		0.633	<0.0001	<0.0001	0.0152	<0.0001

*r*, Pearson correlation coefficient; *p*, significance level; *Z*, value of the Mann–Whitney U test.

There was a statistically significant correlation between nurses’ attitudes toward patient death according to the DAP-R-PL and seniority. Based on the collected data, there was a statistically significant negative correlation between seniority and Fear of death (*p* = 0.02), and a positive correlation between seniority and Approach Acceptance (*p* = 0.0213) (Table 5).

The relationship between nurses’ attitudes toward patient death according to DAP-R-PL and the frequency of respondents’ experience of patient death was shown. A statistically significant negative correlation was found between the frequency of respondents’ experience of patient death and the following attitudes toward death: Death Avoidance (*p* < 0.0001) and Approach Acceptance (*p* = 0.02). In addition, there was a statistically significant positive correlation between the frequency of experiencing a patient’s death and the following attitudes toward death: Natural Acceptance (*p* < 0.0001), Escape Acceptance (*p* < 0.0001) (Table 5).

### 3.3 Effect of stress coping strategies according to MINI-COPE on nurses’ attitudes toward patient death according to DAP-R-PL

The relationship between nurses’ attitudes toward patient death according to DAP-R-PL and choice of stress coping strategies according to MINI-COPE was demonstrated. A statistically significant negative correlation was observed between Fear of death and Active Coping (*p* = 0.0344), as well as a positive correlation between Fear of death and: Self-distraction (*p* < 0.0001), Denial (*p* < 0.0001), Behavioral disengagement (*p* < 0.0001), Venting (*p* < 0.0001), and Self-blame (*p* = 0.0129) (Table 6).

**Table 6:** The relationship between attitudes toward the patient’s death according to DAP-R-PL and the strategy of coping with stress according to MINI-COPE.

MINI-COPE	DAP-R-PL									
	Fear of death		Death avoidance		Natural acceptance		Approach acceptance		Escape acceptance	
	*r*	*p*	*r*	*p*	*r*	*p*	*r*	*p*	*r*	*p*
Active coping	−0.12	0.03	−0.10	0.09	−0.08	0.19	0.17	0.00	−0.15	0.01
Planning	0.02	0.77	−0.06	0.27	0.01	0.92	0.30	<0.0001	0.03	0.63
Positive reframing	−0.02	0.68	−0.01	0.88	−0.07	0.23	0.06	0.31	0.08	0.18
Acceptance	0.04	0.46	0.11	0.05	0.10	0.07	0.20	0.00	0.02	0.75
Humor	−0.04	0.54	0.07	0.20	0.03	0.60	−0.26	<0.0001	0.00	0.97
Religion	0.01	0.87	0.21	0.00	−0.05	0.35	0.65	<0.0001	−0.03	0.55
Use of emotional support	−0.04	0.47	−0.06	0.31	0.05	0.38	0.23	<0.0001	0.09	0.13
Use of instrumental support	0.01	0.88	0.01	0.81	0.03	0.58	0.32	<0.0001	0.08	0.14
Self-distraction	0.25	<0.0001	0.12	0.03	0.11	0.05	−0.31	<0.0001	0.12	0.03
Denial	0.28	<0.0001	0.21	0.00	0.19	0.00	−0.17	0.00	0.25	<0.0001
Venting	0.38	<0.0001	0.22	<0.0001	0.21	0.00	−0.24	<0.0001	0.21	0.00
Substance use	0.08	0.16	−0.21	0.00	0.27	<0.0001	−0.48	<0.0001	0.26	<0.0001
Behavioral disengagement	0.33	<0.0001	0.21	0.00	0.18	0.00	−0.12	0.03	0.16	0.00
Self-blame	0.14	0.01	0.22	<0.0001	−0.10	0.08	0.13	0.03	−0.10	0.07

*r*, Pearson correlation coefficient; *p*, significance level.

Data analysis showed a statistically significant positive correlation between the Death Avoidance attitude and the following stress coping strategies: Acceptance (*p* = 0.0459), Turning to Religion (*p* = 0.0002), Self-distraction (*p* = 0.0275), Denial (*p* = 0.0002), Venting (*p* < 0.0001), Behavioral disengagement (*p* = 0.0002), and a negative correlation with the strategy: Use of Psychoactive Substances (*p* = 0.0003) (Table 6).

In addition, a statistically significant positive correlation was observed between the attitude Naturl Acceptance and the following stress coping strategies: Denial (*p* = 0.0008), Venting (*p* = 0.0002), Use of Psychoactive Substances (*p* < 0.0001), Behavioral disengagement (*p* = 0.0015) (Table 6).

Based on the collected data, there was a statistically significant positive correlation between the Approach Acceptance attitude and the following stress coping strategies: Active Coping (*p* = 0.0030), Planning (*p* < 0.0001), Acceptance (*p* = 0.0005), Turning to Religion (*p* < 0.0001), Use of emotional support (*p* < 0.0001), Use of instrumental support (*p* < 0.0001), and Self-blame (*p* = 0.0260). In addition, there was a statistically significant negative correlation between the attitude Approach Acceptance, and Sense of Humor (*p* < 0.0001), Self-distraction (*p* < 0.0001), Denial (*p* = 0.0033), Venting (*p* < 0.0001), Use of Psychoactive Substances (*p* < 0.0001), Behavioral disengagement (*p* = 0.0311) (Table 6).

The study showed a statistically significant positive correlation between the Escape Acceptance and the following stress coping strategies: Self-distraction (*p* = 0.0345), Denial (*p* < 0.0001), Venting (*p* = 0.0002), Use of Psychoactive Substances (*p* < 0.0001), Behavioral disengagement (*p* = 0.0036), and a negative correlation with: Active Coping (*p* = 0.0072) (Table 6).

## 4 Discussion

Coping with the death of a patient is widely recognized as one of the most emotionally demanding and complex challenges in clinical practice. Patient death affects nurses not only professionally, but also on personal and social levels. Inadequate coping can lead to impaired support for dying patients and their families, as well as increased vulnerability to long-term stress-related consequences.

Our study revealed that the most prevalent attitudes toward death among the surveyed nurses were natural acceptance and escape acceptance. Conversely, the least commonly expressed attitudes included approach acceptance and death avoidance.

A review of the literature indicates that there is no universally dominant attitude toward death among nurses. This finding aligns with the study by Cybulska et al. (21), which investigated 516 Polish nurses and found that natural acceptance was the prevailing attitude, while death avoidance was the least common. Similarly, Makowicz et al. (28), in a study involving 594 nurses, reported that death was primarily viewed as a natural part of life. Fewer respondents described it as a release from suffering or as a transition to a “beyond.” In contrast, Gołębiak et al. (29), in a sample of 115 nurses, found that most participants reported accepting patient death, while others responded with fear, emotional detachment, or avoidance. A smaller subset even denied death’s reality or treated it as unnatural. In another study, Cardoso et al. (12) found that nurses exhibited attitudes such as fear, escape, and avoidance in response to death.

The predominance of natural acceptance in our sample may partly reflect the cultural and religious context of Poland, where the majority of the population identifies as Roman Catholic. Previous studies have shown that higher religiosity can be associated with greater acceptance of death (5, 30). Although religiosity was not assessed in our study, it remains an important factor to consider in future research.

Our findings further demonstrated that attitudes toward death were influenced by sociodemographic and professional variables such as age, gender, place of residence, marital status, professional specialization, and years of experience. Specifically, fear of death was less frequently reported by older nurses and male respondents. Natural acceptance was more common among women, those with advanced education (Master’s/PhD), and those holding a specialization. Additionally, longer professional experience was associated with a lower frequency of fear-based attitudes and a higher occurrence of approach acceptance. The high mean age of our participants is consistent with national statistics for Polish nurses, which likely reduces the risk of selection bias, although it may underrepresent the perspectives of younger professionals.

These patterns are consistent with our earlier research (21), which also found that sociodemographic factors like age, gender, residence, and marital status influence death-related attitudes. Younger nurses more often exhibited death avoidance, while natural acceptance increased with age. Women more frequently expressed natural acceptance, and nurses residing in rural areas were more likely to adopt approach acceptance. Other factors such as the type of patient death, emotional proximity to the patient, level of education, and work experience also shaped these attitudes.

In a study by Gama et al. (31) involving 360 nurses, it was observed that women more often held religious views on death, whereas older and more experienced nurses leaned toward escape acceptance. Oncology and hematology nurses demonstrated higher levels of death avoidance, escape acceptance, and anxiety than their counterparts in palliative care.

Contrastingly, Ponińska et al. (32), in a study of 100 nurses, reported no significant impact of gender, marital status, or education on attitudes toward patient death. However, age and professional seniority significantly influenced these attitudes, with younger nurses struggling more to care for dying patients or to accept death. With age, participants’ value hierarchies shifted.

The predominance of female respondents in our study reflects the demographic structure of the Polish nursing workforce (10). Given that prior research has shown gender differences in death-related attitudes—with women tending to express greater fear of death (33)—this characteristic of our sample should be taken into account when interpreting the results.

Duran et al. (34) found that younger nurses were more likely to demonstrate acceptance of death, whereas single individuals more often adopted escape acceptance. A positive correlation was found between years of experience and both escape and approach acceptance. However, Gołębiak et al. (29) did not find a clear link between seniority and readiness to cope with death, although longer experience correlated with higher empathy levels. Nurses with advanced degrees were more likely to accompany patients at the end of life, while age and workplace setting had no significant influence on emotional responses to death.

Makowicz et al. (28) emphasized that factors such as the patient’s age, type of death, nurse’s seniority, and religious beliefs significantly affected staff attitudes. Less experienced nurses (under five years) and atheists were more likely to distance themselves emotionally from death. Abu Hasheesh et al. (35) also showed that older and more experienced nurses generally held more positive attitudes, while those with limited experience showed more negative ones.

Our own research indicated that the most frequently used stress-coping strategies among nurses included distraction, active coping, planning, and acceptance.

Beyond general recommendations for psychological support, targeted interventions should be aligned with the coping strategies observed in this study. For nurses who predominantly use distraction-oriented coping, mindfulness-based stress reduction and structured leisure activities may reduce stress and prevent emotional exhaustion. For those showing higher levels of acceptance-based coping, psychoeducational workshops and peer-support groups can help maintain resilience and professional engagement. Similar interventions have been shown to reduce burnout and improve psychological well-being among healthcare workers (36, 37).

Statistically significant correlations were found between nurses’ death attitudes (measured via DAP-R-P) and stress coping strategies (measured via MINI-COPE). Supporting this, Sierakowska and Doroszkiewicz (38) reported that during the COVID-19 pandemic, younger nurses more often used strategies such as emotional release, seeking emotional/instrumental support, humor, and self-blame, while nurse managers preferred planning, and hospital nurses demonstrated more effective coping overall. In contrast to our findings, Cardoso et al. reported that Portuguese nurses most frequently demonstrated fear or avoidance attitudes toward death (12). Such differences may be explained by cultural and religious contexts, as Poland is characterized by a predominantly Roman Catholic population (approximately 90%), where religious beliefs and rituals often promote the view of death as a natural transition. Additionally, differences in palliative care training, exposure to end-of-life care, and healthcare system structures may influence how nurses in different countries conceptualize and emotionally respond to death.

Kowalczuk et al. (39) found that Polish and Belarusian nurses most frequently used active coping and planning, and least frequently resorted to substance use or withdrawal. Notably, Polish nurses tended to seek emotional support and used avoidance more often, whereas Belarusian nurses preferred humor. In another study, Bodys-Cupak et al. (40), investigating 394 nursing students, found that those with high self-efficacy were more likely to use active coping, planning, positive reframing, and acceptance, while those with low self-efficacy tended to rely on denial, giving up, and blaming. Studies conducted outside Europe, such as in Japan (41) and Brazil (42), have revealed distinct patterns of death attitudes, often reflecting local religious traditions and societal norms regarding dying and end-of-life care. For example, in Japan, attitudes may be influenced by Buddhist concepts of impermanence, while in Brazil, strong familial involvement and Catholic traditions can shape acceptance or avoidance tendencies. These cross-cultural differences highlight the importance of interpreting our findings within the broader global context and underscore the need for culturally tailored support strategies for nurses.

The relationships between coping strategies, mental health, and burnout are complex. Research shows certain coping styles can mediate or moderate stress and burnout risk (43, 44). Avoidance and rumination worsen stress effects, while problem-focused and emotional support strategies improve well-being (45). Psychological resilience and empathy also buffer occupational stress, reducing burnout (46). Including these factors in models enhances understanding and aids targeted interventions. Theories like Terror Management Theory and Meaning Management Theory explain how facing patient death triggers coping to protect self-integrity and emotional stability (47).

The results of our study can be framed within Lazarus and Folkman (51) stress and coping theory, which posits that an individual’s response to stress depends on their subjective appraisal of the situation and the resources available. Caring for patients at the end of life involves significant emotional burden, requiring coping strategies focused both on the problem and on emotions. The observed differences in attitudes toward death and coping strategies reflect individual variations in stress appraisal and psychological resources. These findings align with concepts of secondary traumatic stress and compassion fatigue (48), which highlight the risk of chronic emotional burden in palliative care. We emphasize the need for systemic psychological support and the development of psychological resilience, consistent with current literature (49, 50). Integrating these theories with our findings strengthens the justification for proposed interventions to support nursing staff.

## 5 Practical implication

The results of the study clearly indicate the need to implement systemic solutions to support nursing staff involved in end-of-life care. Daily exposure to death, suffering, and significant emotional burden is associated with an increased risk of burnout and deterioration in the psychological well-being of nurses. Therefore, it is crucial to develop and implement comprehensive support strategies that adequately address these challenges.

First, we recommend introducing specialized training programs focused on developing adaptive coping skills in response to stress and emotional strain associated with palliative care. Such training should include techniques for emotional regulation, empathetic communication, stress management strategies, and burnout prevention.

Second, we advocate for ensuring regular access to psychological support and clinical supervision for medical personnel, especially those directly engaged in end-of-life care. Access to professional mental health services may reduce the risk of chronic stress, improve interpersonal relationships, and enhance work performance.

Third, it is essential to establish sustainable and accessible organizational support systems—such as debriefing procedures after challenging clinical cases, flexible employment arrangements, and institutional mechanisms for reporting psychological overload. Institutional support should be tailored to the realities of nursing work and responsive to the staff’s actual needs.

It is also important that when designing interventions and systemic solutions, sociodemographic differences (such as age, gender, level of education, and workplace) and cultural context are taken into account, as these factors may influence both attitudes toward death and coping strategies.

Implementing these recommendations could significantly improve the psychological and occupational well-being of nursing staff, enhance the quality of care provided to patients at the end of life, and reduce professional turnover in this critical healthcare workforce group.

## 6 Limitation

The present study has several limitations. First, the sample size and random selection of participants may limit the representativeness of the findings. In addition, the sample was characterized by a pronounced gender imbalance, with 85.7% of participants being women. This reflects the high feminization of the nursing profession in Poland (10), yet it may also have influenced the results, as previous research indicates that gender can shape attitudes toward death, with women often reporting higher levels of death anxiety (33).

Another limitation concerns the absence of several potentially relevant variables, including religious beliefs, personal experiences of loss, and severity of stress. Prior studies have demonstrated that religiosity is associated with attitudes toward death, particularly approach and natural acceptance (5, 30). These factors were omitted due to questionnaire length constraints and the decision to prioritize coping strategies. Nonetheless, their exclusion restricts the comprehensiveness of the analysis and highlights the need for their inclusion in future research.

The relatively high mean age of participants mirrors the demographic profile of the Polish nursing workforce, where the average age exceeds 50 years (10). While this reflects professional reality, it may limit the generalizability of the results to younger nurses. Moreover, the inclusion of nurses exclusively from hospital settings further restricts the applicability of findings to those employed in community or primary care environments.

Finally, the cross-sectional design of the study prevents assessment of temporal changes and causal relationships. Longitudinal research is warranted to capture the dynamics of attitudes toward death and coping strategies over time. Such approaches could provide deeper insight into the psychological and professional challenges nurses encounter in end-of-life care.

## 7 Conclusion

Nurses show different attitudes toward the death of a patient, but most often they choose natural acceptance of death and escape acceptance of death. Both age, gender, marital status and place of residence significantly influenced nurses’ attitudes toward patient death according to the DAP-R-PL. Also, education, having a specialty, and seniority and place of work determined nurses’ attitudes toward patient death.

The study results indicate the need to continue exploring nurses’ attitudes toward patient death and the coping strategies they employ, using research approaches that allow for the analysis of changes over time. The application of longitudinal studies would enable a deeper understanding of the dynamics of these phenomena and their relationship with personality, environmental, and professional variables. These analyses should be complemented by qualitative research, which can provide an in-depth insight into the individual experiences of those working in end-of-life care, as well as into their broader psychosocial context. The inclusion of both quantitative and qualitative approaches may contribute to a more comprehensive understanding of the issue and support the development of more targeted and effective interventions for healthcare professionals.

## Data Availability

The raw data supporting the conclusions of this article will be made available by the authors, without undue reservation.
